# Comparative Analysis of Adipose-Derived Stromal Cells and Their Secretome for Auricular Cartilage Regeneration

**DOI:** 10.1155/2020/8595940

**Published:** 2020-02-03

**Authors:** Se-Joon Oh, Kyung-Un Choi, Sung-Won Choi, Sung-Dong Kim, Soo-Keun Kong, Seokwhan Lee, Kyu-Sup Cho

**Affiliations:** ^1^Department of Otorhinolaryngology and Biomedical Research Institute, Pusan National University Hospital, Busan, Republic of Korea; ^2^Department of Pathology, Pusan National University School of Medicine, Pusan National University Hospital, Busan, Republic of Korea

## Abstract

Adipose-derived stromal cells (ADSCs) can repair auricular cartilage defects. Furthermore, stem cell secretome may also be a promising biological therapeutic option, which is equal to or even superior to the stem cell. We explored the therapeutic efficacies of ADSCs and their secretome in terms of rabbit auricular cartilage regeneration. ADSCs and their secretome were placed into surgically created auricular cartilage defects. After 4 and 8 weeks, the resected auricles were histopathologically and immunohistochemically examined. We used real-time PCR to determine the levels of genes expressing collagen type II, transforming growth factor-*β*1 (TGF-*β*1), and insulin-like growth factor-1 (IGF-1). ADSCs significantly improved auricular cartilage regeneration at 4 and 8 weeks, compared to the secretome and PBS groups, as revealed by gross examination, histopathologically and immunohistochemically. ADSCs upregulated the expression of collagen type II, TGF-*β*1, and IGF-1 more so than did the secretome or PBS. The expression levels of collagen type II and IGF-1 were significantly higher at 8 weeks than at 4 weeks after ADSC injection. Although ADSCs thus significantly enhanced new cartilage formation, their secretome did not. Therefore, ADSCs may be more effective than their secretome in the repair of auricular cartilage defect.

## 1. Introduction

Auricular cartilage has a low intrinsic capacity for repair because it is not vascularized; defects or tears may trigger severe deformity and disfigurement [1]. Cartilage tissue engineering may overcome the limitations of autologous cartilage grafting, reestablishing the unique biological and functional properties of cartilage [2]. Mesenchymal stem cells (MSCs) can differentiate into several mesenchymal lineages, such as those forming bone [3–5], adipose tissue [3, 4], and cartilage [3, 4]; recent studies have focused on MSC differentiation into chondrocytes to aid in cartilage tissue engineering [6]. Rabbit auricular cartilage defects were completely repaired by chondrocytes derived from allogeneic bone marrow-derived MSCs [7]. Adipose-derived stromal cells (ADSCs) also repaired such defects, associated with high-level expression of the proteins S-100, collagen type II, and transforming growth factor-*β* (TGF-*β*) [8]. Recently, stem cell-derived substrates and secretomes have been used as therapeutic tools and for cartilage regeneration [1]. Several studies have shown that the therapeutic efficacy of stem cell transplantation is attributable to multiple secreted factors that regulate the surrounding microenvironment to evoke regeneration via a paracrine mechanism [9–11]. MSCs secrete an array of growth factors, cytokines, and extracellular vesicles; these enhance collagen type II expression and downregulate matrix metalloproteinase expression in osteoarthritic chondrocytes [12]. Although stem cells and their secretomes afforded similar therapeutic benefits in terms of animal articular cartilage regeneration [13], no study has yet compared the effects of stem cells and their secretomes on the repair of auricular cartilage defect.

The purpose of the present study was to explore whether ADSCs and their secretome promoted cartilage repair and to compare the therapeutic efficacy for cartilage regeneration between ADSCs and secretome in animal models of auricular cartilage defects.

## 2. Materials and Methods

### 2.1. Animals

Six-month-old female Dutch rabbits were purchased from Samtako Co. (Osan, Republic of Korea, http://www.samtako.co.kr) and bred in a specific pathogen-free facility. The study protocol was approved by the Institutional Animal Care and Use Committee of Pusan National University School of Medicine (no. PNUH-2015-075).

### 2.2. ADSC Isolation and Culture

As described previously [8], adipose tissue was obtained from the inguinal region, washed extensively with equal volumes of phosphate-buffered saline (PBS), and digested with 0.075% collagenase type I (Sigma, St. Louis, MO, USA) at 37°C for 30 min. After neutralization of enzyme activity with *α*-modified Eagle's medium (*α*-MEM) containing 10% fetal bovine serum (FBS), the samples were centrifuged at 1,200 × g for 10 min and the pellets were incubated overnight in control medium at 37°C under 5% CO_2_. Nonadherent red blood cells were removed, and third- or fourth-passage adherent ADSCs were used after phenotypic characterization [14].

Flow cytometric analysis was used to characterize the phenotypes of the ADSCs. At least 50,000 cells were incubated with fluorescein isothiocyanate-labeled monoclonal antibodies against mouse stem cell antigen-1, CD44, CD45, CD117, and CD11b (Clontech, BD Biosciences, Palo Alto, CA) or with the respective isotype control. After washing, labeled cells were analyzed by flow cytometry using a FACSCalibur flow cytometer and Cell-Quest Pro software (BD Biosciences, San Diego, CA). The expression percentage of each marker of ADSCs was determined by the percentage of positive events, as determined compared to the isotype-matched negative control.

ADSCs were analyzed for their capacity to differentiate into adipogenic, osteogenic, and chondrogenic lineages, as described previously [14]. For adipogenic and osteogenic differentiation, cells were seeded in 6-well plates at a density of 20,000 cells/cm^2^ and treated for 3 weeks with adipogenic and osteogenic media. Adipogenic and osteogenic differentiation was assessed using Oil Red O staining, as an indicator of intracellular lipid accumulation, and Alizarin Red S staining, as an indicator of extracellular matrix calcification, respectively. Chondrogenic differentiation was induced using the micromass culture technique. Briefly, 10 mL of a concentrated ADSC suspension (3 × 10^5^ cells/mL) was plated in the center of each well and treated for 3 weeks with chondrogenic medium. Chondrogenesis was confirmed by immunohistochemistry.

### 2.3. ADSC Labeling

ADSCs were washed with PBS and incubated with 2 *μ*M Cell Tracker CM-Dil (Molecular Probes Inc., Eugene, OR) at 37°C for 5 min and then for an additional 15 min at 4°C. The cells were washed with PBS and suspended in PBS at 2 × 10^7^ cells/mL. CM-Dil-labeled ADSCs were observed under a fluorescence microscope (Olympus, Tokyo, Japan; model IX7122FL/P) at an excitation wavelength of 553 nm.

### 2.4. Isolation of the ADSC Secretome

The supernatants of ADSC cultures (the secretome) were pressure-concentrated (ca. 50-fold) (Amicon, Millipore Corp., Billerica, MA, USA) using a 3000 Da pore-sized membrane. Salts were eliminated employing a HiTrap Desalting kit (GE Healthcare, Uppsala, Sweden). Lipopolysaccharide (LPS) was depleted (endotoxin level < 0.01 *μ*g/mL) using Detoxi-Gel Affinity Pak prepacked columns (Pierce, Rockford, IL) in accordance with the manufacturer's instructions.

### 2.5. Subperichondrial Injection of ADSCs and the Secretome

**Figure 1:**
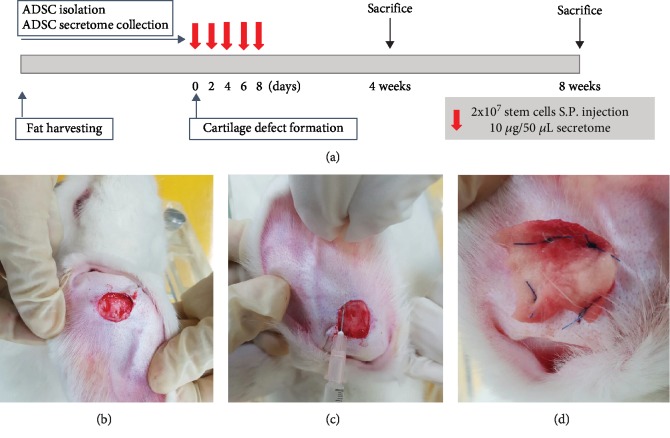
The experimental protocol and method. (a) Purified adipose-derived stromal cells (ADSCs; 2 × 10^7^ cells/mL) or 10 *μ*g/50 *μ*L of the secretome was injected subperichondrially (S.P.) into 2 cm diameter, surgically created, auricular cartilage defects (b) on days 0, 2, 4, 6, and 8. The materials were injected (using a 26-gauge needle) at the edge of the excision site (c), and Spongostan was sutured to each defect (d).

The experimental protocol is summarized in Figure 1(a). Twenty Dutch rabbits were anesthetized with 2% xylocaine (1 mg/kg) and alfaxan (5 mg/kg). After shaving and povidone iodine dressing, we removed a spherical 20 × 20 mm cartilage plate involving both the perichondrium and skin from the midportion of each auricle, leaving the outer skin intact (Figure 1(b)). The right ears were treated with ADSCs (*n* = 10) or the secretome (*n* = 10) and the left ears (*n* = 20) with PBS. A total of 2 × 10^7^ purified ADSCs or 10 *μ*g/50 *μ*L of the secretome was injected (using a 26-gauge needle) under the perichondrium, at six points around the edge of the excision site, on postoperative days (PODs) 0, 2, 4, 6, and 8 (Figure 1(c)). All defects were covered with Spongostan (Ferrosan, Copenhagen, Denmark) to prevent dehydration; normal saline was applied daily to maintain moistness (Figure 1(d)).

### 2.6. Auricular Cartilage Regeneration

All ears were regularly inspected. Photographs were taken preoperatively, immediately postoperatively, and weekly thereafter; we measured the defect size and noted any inflammatory change. The defect size was the mean of the major and minor axes. All assessments were performed by an investigator blinded to treatment details.

### 2.7. Histology and Immunohistochemistry

Five of 10 rabbits were sacrificed 4 weeks after ADSC or secretome injection, and the remaining rabbits sacrificed at 8 weeks. No animal died prior to sacrifice. After intraperitoneal phenobarbital injection (80 mg/kg), the auricles were resected and sent for histopathological examination. Sections were obtained from paraffin wax-embedded specimens and stained with hematoxylin and eosin (H&E). Elastin immunohistochemistry was performed to evaluate new cartilage formation and the extent of cartilage maturation, as previously described [15]. To detect elastin, sections were incubated with a polyclonal rabbit anti-elastin antibody (#RDI-TP 592; Research Diagnostics Inc., Flanders, NJ) at a dilution of 1 : 000 (Dako Z0311) at room temperature for 2 h and stained with Polymer-HRP (Dako EnVision®+ Dual Link System-HRP (DAB+) K4065) for 30 min. Negative controls did not receive the primary antibody.

### 2.8. Quantitative Real-Time Reverse Transcription Polymerase Chain Reaction

Prior to quantitative real-time polymerase chain reaction (qRT-PCR), total RNAs were extracted and reverse-transcribed using random hexamers, as previously described [10]. RT-PCR was performed using 10 ng of cDNA/tube and SYBR Green Mix (Bio-Rad Laboratories, Hercules, CA). Transcript-specific primers for collagen type II (NM_001844), IGF-1 (NM_000618), and TGF-*β*1 (NM_000660) were designed based on the GenBank cDNA sequences of Table 1. Expression levels are presented as -fold increases compared to those of glyceraldehyde-3-phosphate dehydrogenase (GAPDH), using the formula 2^(ΔCt)^, where ΔCt = Ct of the target gene minus Ct of GAPDH (NM_002046) [16].

### 2.9. Statistical Analysis

All experiments were repeated at least three times. Data are presented as means ± standard errors of the means. Statistical analysis featured Kruskal-Wallis testing followed by the Bonferroni-corrected, two-group, and post hoc Mann-Whitney *U* test for continuous variables. We employed IBM SPSS Statistics for Windows ver. 22.0 software (IBM Corp., Armonk, NY). A *p* value < 0.05 was considered to indicate statistical significance.

## 3. Results

### 3.1. Characterization of the ADSC Immunophenotype and ADSC Differentiation

Cultured ADSCs were negative for the cell surface markers CD45, CD117, and CD11b, but positive for Sca-1, CD44, and CD90. The ADSCs were spindle- shaped (thus, fibroblast-like), as in previous reports on ADSCs and bone marrow-derived MSCs. The ADSCs could differentiate into adipogenic, osteogenic, and chondrogenic lineages after culture under appropriate conditions (Supplementary [Supplementary-material supplementary-material-1]).

### 3.2. Gross Findings

**Figure 2:**
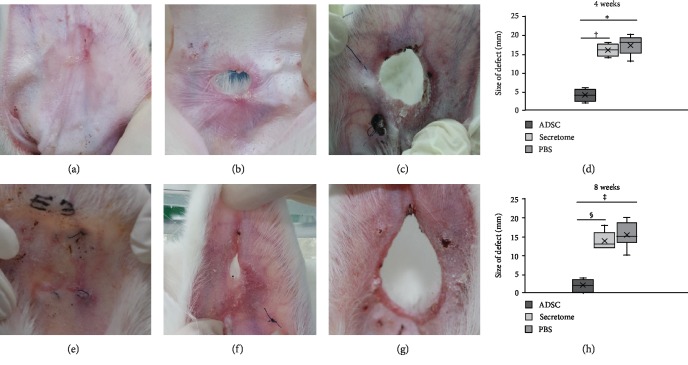
Gross auricular cartilage findings. (a) Four weeks after ADSC injection, the defects were filled with soft pinkish tissue. The defects of the secretome (b) and PBS (c) groups remained evident. The defect size decreased significantly in the ADSC compared to the secretome or the PBS group at 4 (d) and 8 weeks (H) after ADSC injection. (e) In ADSC-treated ears, the defects were completely healed by 8 weeks; the tissue surface was smooth and of similar color to that of the surrounding tissue. The secretome (f) and PBS (g) groups exhibited minimal defect repair. ^∗^^,†,‡,§^*p* < 0.001. ADSCs: adipose-derived stromal cells; PBS: phosphate-buffered saline.

No auricle exhibited any sign of inflammation (skin erythema or discharge). In the ADSC group, three of 10 defects were completely repaired, exhibiting smooth, mildly pink surfaces, 4 weeks after injection (Figure 2(a)). Furthermore, 8 weeks after injection, all defects except one were completely healed, thus, similar in color to the surrounding tissue (Figure 2(e)). In the secretome group, no obvious healing was evident 4 weeks after injection (Figure 2(b)). Although the defect size at 8 weeks post-injection was somewhat decreased (Figure 2(f)), such reduction was also noted in the PBS group (Figures 2(c) and 2(g)). The defect sizes 4 weeks postinjection were 4.0 ± 1.6, 16.0 ± 1.6, and 18.2 ± 2.9 mm in the ADSC, secretome, and PBS groups, respectively; the 8-week figures were 1.8 ± 1.8, 13.8 ± 2.5, and 16.6 ± 4.2 mm. The ADSC group evidenced much better auricular cartilage regeneration (compared to the secretome or PBS group) (*p* < 0.001) at both 4 (Figure 2(d)) and 8 (*p* < 0.001) weeks (Figure 2(h)). However, the secretome and PBS groups did not differ.

### 3.3. Detection of ADSCs in Auricular Cartilage Defects

**Figure 3:**
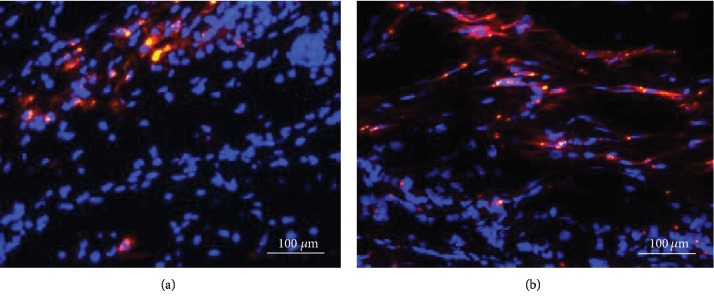
Immunofluorescence of adipose-derived stromal cells (ADSCs) in auricular cartilage defects. ADSCs labeled with the red Cell Tracker CM-Dil dye were detected in the ADSC group at 4 (a) and 8 weeks (b) after ADSC injection.

Immunofluorescence microscopy revealed red (CM-Dil-positive) ADSCs at the sides of the defects 4 and 8 weeks after ADSC injection (Figures 3(a) and 3(b)). The ADSCs had integrated with the surrounding tissues at 8 weeks.

### 3.4. Microscopic Findings and Immunohistochemistry

**Figure 4:**
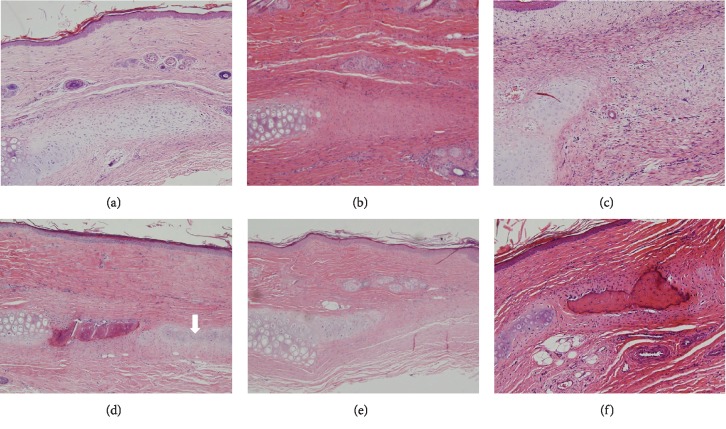
Histopathological features of auricular cartilage defects at 4 (a–c) and 8 (d–f) weeks after adipose-derived stromal cell (ADSC) injection. Chondroblasts and cartilage-specific extracellular matrix (ECM) were evident in the ADSC group (a). ECM was deposited in the secretome group (b), and fibrous tissue formation was apparent in the PBS group (c). Typical cartilaginous features (chondrocytes, chondroblasts, and cartilage-specific ECM (arrow)) were observed in the ADSC group (d). Small numbers of chondroblasts and a little collagenous tissue were evident at the defect edges of the secretome (e) and PBS groups (f), respectively. The sections were stained with hematoxylin and eosin (magnification 200x).

**Figure 5:**
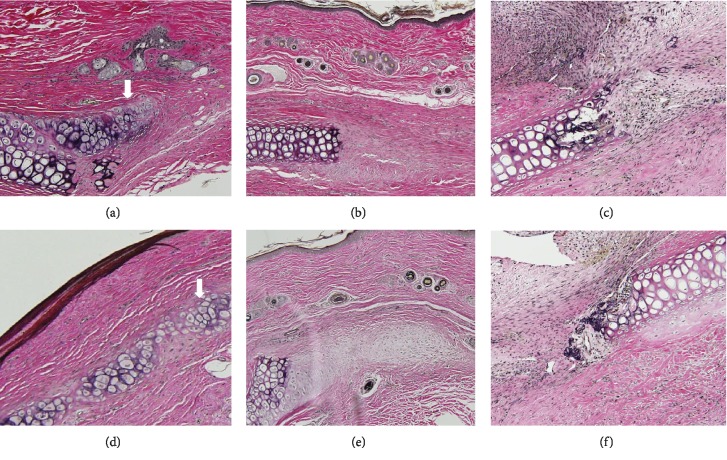
Immunohistochemical detection of elastin. New cartilage (arrow) was evident in the adipose-derived stromal cell (ADSC) group at 4 (a) and 8 weeks (d) after ADSC injection. Small amounts of ECM and fibrous tissue were observed, respectively, in the secretome (b, e) and PBS (c, f) groups at 4 and 8 weeks (magnification, 200x).

Four weeks after ADSC injection, typical cartilage featuring chondrocytes, chondroblasts, and cartilage-specific extracellular matrix (ECM) was evident (Figure 4(a)). No new cartilage formation was apparent in the secretome group or PBS group, although fibrous tissue was observed on H&E staining (Figures 4(b) and 4(c)). At 8 weeks postinjection, the ADSC group contained mature cartilage with obvious lacunae and a dense ECM filling any cartilage defects (Figure 4(d)). However, only minimal chondrocyte proliferation was detected in the secretome and PBS groups (Figures 4(e) and 4(f)). Elastin immunohistochemistry revealed new cartilage at 4 and 8 weeks after ADSC injection (Figures 5(a) and 5(d)). Although some ECM was observed in the secretome group (Figures 5(b) and 5(e)), only fibrous tissue was detected in the PBS group at 4 and 8 weeks postinjection (Figures 5(c) and 5(f)).

### 3.5. Expression of Collagen Type II and Growth Factor Genes

**Figure 6:**
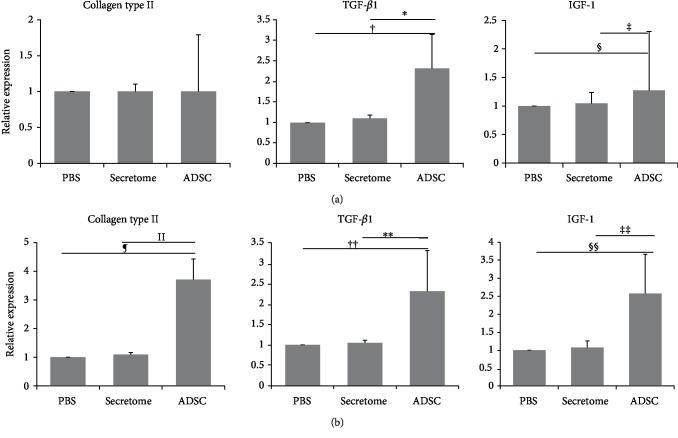
The expression levels of genes encoding collagen type II and various growth factors at 4 (a) and 8 (b) weeks after ADSC injection. The collagen type II expression level was significantly higher in the ADSC than in the secretome or PBS group 8 weeks after ADSC injection. In the ADSC group, the expression levels of transforming growth factor- (TGF-) *β*1 and insulin-like growth factor (IGF) were significantly higher than in the secretome or PBS group at 4 and 8 weeks postinjection. ^∗^^,‡‡^*p* = 0.002; ^†,§§^*p* = 0.001; ^‡^*p* = 0.023; ^§^*p* = 0.011; ^ǁ,¶,^^∗∗^^,††^*p* < 0.001. ADSCs: adipose-derived stromal cells; PBS: phosphate-buffered saline.

At 4 weeks after ADSC injection, the relative expression levels of collagen type II, IGF-1, and TGF-*β*1 (compared to the PBS group) were 1.00 ± 0.02, 1.27 ± 0.32, and 2.33 ± 0.57 in the ADSC group and 1.00 ± 0.06, 1.05 ± 0.07, and 1.03 ± 0.08 in the secretome group. At 8 weeks, the figures were 3.70 ± 0.74, 2.57 ± 0.75, and 2.32 ± 0.64 in the ADSC group and 1.09 ± 0.10, 1.07 ± 0.06, and 1.10 ± 0.12 in the secretome group. The expression levels of IGF-1 and TGF-*β*1 in the ADSC group were significantly increased at 4 weeks compared to the secretome (*p* = 0.04 and *p* = 0.008, respectively) and PBS (*p* = 0.008 and *p* = 0.008, respectively) groups (Figure 6(a)). The expression levels of collagen type II, IGF-1, and TGF-*β*1 in the ADSC group were significantly increased at 8 weeks compared to the secretome (*p* < 0.001, *p* = 0.002, and *p* < 0.001, respectively) and PBS (*p* < 0.001, *p* = 0.001, and *p* < 0.001, respectively) groups (Figure 6(b)). Notably, the expression levels of collagen type II and IGF-1 were significantly higher at 8 than 4 weeks after ADSC injection (*p* = 0.008 and *p* = 0.016, respectively). However, no significant difference in the expression level of any of collagen type II, IGF-1, or TGF-*β*1 was evident between the secretome and PBS groups.

## 4. Discussion

Stem cell therapy is promising in terms of cartilage regeneration; such cells can differentiate into several lineages [17]. The therapeutic effects of transplanted MSCs are supposed to reflect MSC migration to the site of injury, cell integration into damaged tissue, and differentiation into specialized tissue [18]. MSCs secrete many autocrine or paracrine factors including growth factors, cytokines, and chemokines (the secretome), creating a microenvironment supporting MSC cell survival, renewal, and differentiation, as well as modulating inflammatory reactions and inducing angiogenesis, culminating in regeneration [19, 20]. Many studies have shown that the secretome plays important roles in regeneration of cardiovascular [21], liver [22], lung [23], and renal [24] injuries. Stem cell-conditioned medium has been used to heal wounds in many preclinical studies and serves as an acceptable alternative to cell therapy [25–27]. This has encouraged the use of the stem cell secretome to accelerate auricular cartilage regeneration. Here, we compared the regenerative effects of ADSCs and their secretome on auricular cartilage defects; we performed gross observations, histological analysis, immunohistochemistry, and qRT-PCR. ADSCs accelerated auricular cartilage repair, more so than did the secretome or PBS. ADSCs labeled with CM-Dil were observed in the defects 4 and 8 weeks postinjection, suggesting that the ADSCs played an important role in cartilage regeneration. These findings were histologically confirmed; new cartilage featuring chondrocytes and cartilage-specific ECM was evident in the defects. The ADSCs acquired a chondroblastic phenotype, expressing elastin strongly, as evidenced by both immunohistochemical staining and higher-level expression of type II collagen on qRT-PCR.

Many factors have been implicated in cell recruitment, proliferation, survival, and differentiation during cartilage regeneration. Of the various cytokines and growth factors, the TGF-*β* superfamily contains well-established growth factors used to induce chondrogenic differentiation [8]. TGF-*β*1 is involved in classical chondrogenic differentiation and stimulates the synthetic activities of chondrocytes [28]. IGF-1 is required principally to maintain cartilage integrity and facilitate cartilage maturation by increasing the production of sulfated glycosaminoglycans and retaining these materials within the pericellular matrix [29]. We found that ADSC transplantation significantly increased TGF-*β*1 and IGF-1 expression. However, such expression did not differ significantly between the secretome and PBS groups. The auricle is a plate of elastic cartilage; the predominant collagen is of type II [30], and the expression of which usefully confirms the chondrocyte phenotype. Collagen II expression was significantly increased in the ADSC compared to the secretome and PBS groups. Taken together, the data indicate that ADSCs per se promoted cartilage regeneration in terms of chondrocyte differentiation and maturation and ECM synthesis. The higher-level expression of collagen type II and IGF-1 at 8 rather than 4 weeks postinjection may indicate that chondrocytes mature in terms of ECM production over time.

A recent systematic review/meta-analysis found that the secretome afforded a therapeutic benefit similar to that of stem cell transplantation in terms of articular cartilage regeneration in animal models [13]. However, we found that the ADSC secretome had no significant impact on auricular cartilage regeneration. These contradictory results are difficult to explain but may be attributable to differences in the surrounding microenvironments. Articular cartilage is a hyaline cartilage lining the articular surfaces of bone ends in the articulating joint (an enclosed space). Therefore, secretome transplantation may play a pivotal role in articular cartilage regeneration. However, in our present study, the ADSC secretome could not be retained in long term in the open, surgically created, auricular cartilage defect.

Our study has some limitations. It is unclear whether ADSCs injected into a surgically created auricular cartilage defect directly differentiated into chondrocytes. Further studies are required to fully characterize the regenerative effects of ADSCs on auricular cartilage defect by repeating our experiments with lineage tracing.

## 5. Conclusion

ADSCs have beneficial effects, but secretome has no significant impact on the auricular cartilage regeneration. Therefore, ADSCs might be more effective treatment than their secretome in the repair of auricular cartilage defect.

## Figures and Tables

**Table 1:** Reverse transcription-polymerase chain reaction primer sequences.

Primer name	Sequence (5′-3′)	Size (bp)	Gene accession no.
GAPDH	Sense: TCGACAGTCAGCCGCATCTTCTTT	94	NM_002046
	Antisense: ACCAAATCCGTTGACTCCGACCTT		

Collagen type II	Sense: CCCTGAGTGGAAGAGTGGAG	511	NM_001844
	Antisense: GAGGCGTGAGGTCTTCTGTG		

IGF-1	Sense: AGGAAGTACATTTGAAGAACGCAAGT	103	NM_000618
	Antisense: CCTGCGGTGGCATGTCA		

TGF-*β*1	Sense: GGCAGTGGTTGAGCCGTGGA	590	NM_000660
	Antisense: TGTTGGACAGCTGCTCCACCT		

GAPDH: glyceraldehydes-3-phosphate dehydrogenase; IGF: insulin-like growth factor; TGF: transforming growth factor.

## Data Availability

The data used to support the findings of this study are available from the corresponding author upon request.

## Supplementary Materials

Supplementary Materials Supplementary Figure 1. Characteristics of adipose-derived stromal cells (ADSCs). ADSCs showed characteristics of mesenchymal stem cells in the fibroblast-like morphology appearance (a), osteogenesis (b), chondrogenesis (c), and adipogenesis (d) (magnification, ×100).
