# Supplementary material for: Comparative Analysis of Adipose-Derived Stromal Cells and Their Secretome for Auricular Cartilage Regeneration
Source: Stem Cells Int. 2020 Feb 3;2020:8595940. doi: 10.1155/2020/8595940 (PMC7023823; doi:10.1155/2020/8595940)
Supplement: Supplementary Materials — Supplementary Figure 1. Characteristics of adipose-derived stromal cells (ADSCs). ADSCs showed characteristics of mesenchymal stem cells in the fibroblast-like morphology appearance (a), osteogenesis (b), chondrogenesis (c), and adipogenesis (d) (magnification, ×100). [file 8595940.f1.docx]

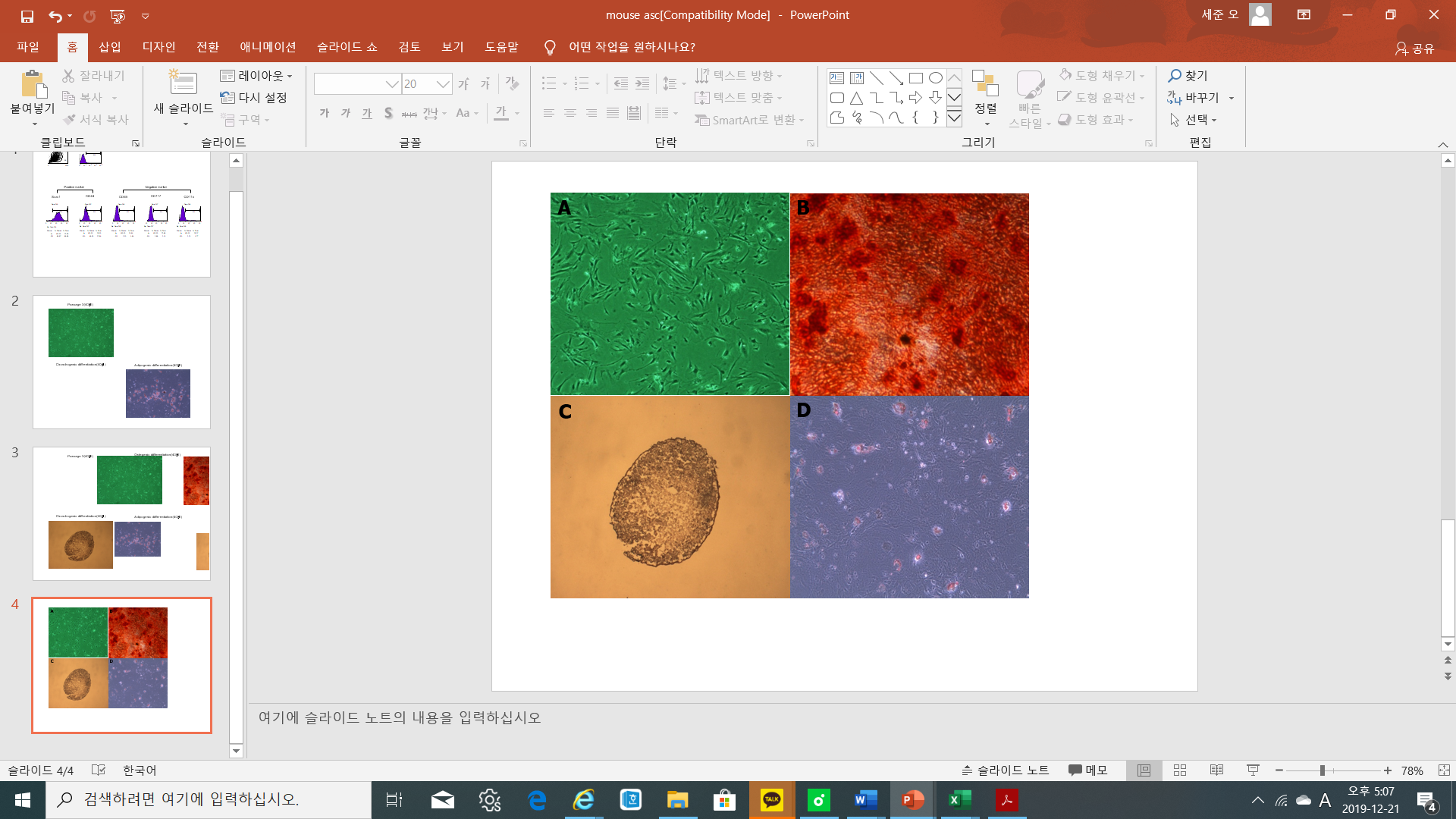


Supplementary Figure 1. Characteristics of adipose-derived stromal cells (ADSCs). ADSCs showed characteristics of mesenchymal stem cells in the fibroblast-like morphology appearance (a), osteogenesis (b), chondrogenesis (c), and adipogenesis (d) (magnification, x100).
